# Opioid deaths in children in Ontario: A province-wide study

**DOI:** 10.1093/pch/pxae064

**Published:** 2024-10-10

**Authors:** Katrina Assen, Elizabeth Urbantke, Michael Wilson, Michael Rieder

**Affiliations:** Department of Paediatrics, Schulich School of Medicine & Dentistry, University of Western Ontario; Office of the Chief Coroner of Ontario; Thunder Bay Regional Health Sciences Centre, Thunder Bay, Ontario, Canada; Department of Paediatrics, Schulich School of Medicine & Dentistry, University of Western Ontario

**Keywords:** *Child protection*, *Children*, *Fentanyl*, *Opiates*, *Toxicity*

## Abstract

**Objectives:**

Opioid-related deaths are an ongoing concern. There have been increasing numbers of fentanyl-related adult deaths with limited knowledge of the characteristics and circumstances of opioid toxicity deaths in children. Our aim was to address this using province-wide data capturing all deaths in children under the age of 10 years in Ontario.

**Methods:**

Data were extracted from the opioid investigative aid database at the Office of the Chief Coroner from the implementation of the system from October 1, 2017, to October 31, 2021. This collects all opioid-related deaths in Ontario (population 14.7 million). A chart review was undertaken on all deaths under 10 years of age. Patient characteristics were calculated as percentages; descriptive analysis was conducted.

**Results:**

Ten deaths in children under the age of 10 occurred during the study period. The average age was 1.9 years with the oldest being 4 years and 9 months. The causative opioid was fentanyl alone in four cases (40%), fentanyl and other drugs in four cases (40%), and hydromorphone and methadone in one case each (10%). Most cases involved improperly stored medication or illicit substances. All children who died had previous child protection service involvement, and at least 70% of their families had previous police involvement.

**Conclusions:**

Fentanyl was the primary substance involved in 80% of deaths. Several potential areas of system change include education on fentanyl risk to young children, careful storage of illicit substances, and implications for how the child protection system intervenes in homes where the use of opioids and illicit substance use is reported to occur.

The opioid epidemic is a public health crisis across Canada and the USA. With the impact of fentanyl, an ongoing rise in opioid-related mortality has been seen in Canada since 2016. There were an estimated 8 deaths per day in 2016, which increased to 12 in 2018 and 20 in 2022 (1). Despite the concern of a public health crisis with escalating numbers, the impact that the opioid crisis has had on young children has received less consideration. Many of these substances or drug mixtures associated with death contain fentanyl, which can cause fatal toxicity with relatively small amounts ingested. A recent American study examining trends of overdose mortality in those less than 12 years of age from 1999 to 2018 found only 11.4% were secondary to synthetic opioids such as fentanyl (2). However, this is an understudied issue in children and multiple studies have either excluded illicit opioids or did not report their incidence (3,4). Of note, when data from 1999 to 2021 were reviewed, the number of children’s deaths in overdose settings associated with opiates increased to approximately 33% (5). Thus, we are likely greatly underestimating the present-day effect the opioid epidemic has on child health.

One of the most important groups of children to target is those less than 10 years of age. Given that 75% to 92% of opioid toxicity in this age group is unintentional, this toxicity is often entirely preventable (4,6). A detailed understanding of the circumstances surrounding these deaths is required to better guide public policy and the health care system response. There have been several studies describing specific cases of opioid-related deaths in children (7,8); however, none have been in Canada, and many are not reflective of current opioid use. As of 2017, the Office of the Chief Coroner of Ontario has been specifically collecting data on all opioid-related deaths in Ontario affording a unique opportunity to provide insight into pediatric-related mortality on a population-wide basis. We sought to examine cases of opioid deaths in children less than 10 years of age and use situational descriptions to better the potential for intervention.

The objectives of the study were threefold. To characterize the demographics of children under 10 with death due to opioid toxicity in Ontario. Secondly, to describe the circumstances surrounding opioid toxicity and third, to identify potential areas for public policy intervention to reduce fatal opioid toxicity in children under age 10.

## METHODS

The Office of the Chief Coroner of Ontario is the largest centralized coroner system in North America which investigates all non-natural deaths, including all deaths due to drug toxicity. It serves the province of Ontario with a population in 2021 of 14.7 million (9). All coroners in Ontario are physicians with specialized training in death investigation. In 2017 the Opioid Investigative Aid (OIA) database was created by the Office of the Chief Coroner to facilitate the examination of all suspected opioid-related deaths and to collect detailed information regarding these deaths.

Anonymized data for all opioid-related deaths in children aged 10 and under was collected from the OIA database dating from October 2017, when the current system was implemented, to October 31, 2021. Pursuant to Section 10 of the Coroners Act all deaths in Ontario under these circumstances are investigated by a Coroner with toxicological analysis conducted at the Ontario Centre of Forensic Science in Toronto. Information collected includes demographics, situational details surrounding the death, and drug toxicity data. Patient characteristics were calculated as percentages and descriptive analysis was conducted. Data was extracted and reviewed by a pediatrician and an investigating coroner.

This project was approved by the Research Ethics Board at the University of Western Ontario and by the Office of the Chief Coroner. Given the nature of the study, the Research Ethics Board approved a waiver of parental consent.

## RESULTS

Ten childhood deaths occurred during the study period. The average patient age was 1.9 years with the oldest child being 4 years and 9 months and the youngest being 9 months (Table 1). Six children were male, seven were white, and three were Indigenous. Three children had chronic medical conditions, one with multiple congenital anomalies, another with developmental delay with possible autism, and lastly a child with an asymptomatic ventricular septal defect. Of note, none of these children were receiving prescription medications at the time of death.

**Table 1.** Patient characteristics

	No. (%)
Total patients	10
Median age (years)	1.89
Sex	
Male	6 (60%)
Race	
White	7 (70%)
Indigenous	3 (30%)
Number of people in dwelling, average	4.67
Previously healthy	7 (70%)
Drug-associated material at the scene	8 (80%)
Previous involvement with Child Welfare Services	10 (100%)
Previous police involvement	
Yes	7 (70%)
No	1 (10%)
Unknown	2 (20%)
Place of death	
Home	8 (80%)
Relative’s Home	1 (10%)
Inpatient	1 (10%)
Type of opiate toxicity	
Fentanyl combination	4 (40%)
Fentanyl alone	4 (40%)
Methadone	1 (10%)
Hydromorphone	1 (10%)
Manner of Death*	
Undetermined	5 (50%)
Accident	5 (50%)

*Manner of Death is the broad category by which death occurrs and includes Natural, Accident, Suicide, Homicide, and Undetermined. In the case of Undetermined, it is not clear if the death occurred by one of the other four Manners

Among the parents six had reports of recreational drug use by their parents and four of the cases lived in households where a parent was prescribed opioids. Two of the deaths were due to prescribed opioids (one each of methadone and hydromorphone). Five of the 10 homes had parents who smoked cigarettes (unknown for 2 others). All children had previous child welfare service (CWS) involvement and 7 of 10 came from households with previous police involvement (unknown for two other homes). The average number of people per household was 4.7.

Detailed descriptions were included of the scene where the child was found dead (Table 2). Eight occurred in their home, one in a relative’s home, and one as an inpatient in a hospital. Eight had drug-associated material at the scene and in four deaths alcohol was present in the home. The causative opioid was hydromorphone (N = 1), methadone (N = 1), fentanyl alone (N = 4), and fentanyl in combination (N = 4). Typically, fentanyl was in combination with etizolam, though one case had morphine and cocaine as secondary drugs.

**Table 2.** Detailed case information

Case	Primary opioid toxicity	Opioid concentration	Other drugs	Circumstances
1	Hydromorphone	60 ng/mL	None	Parent prescribed hydromorphone. Was febrile the night prior. Initially slept in their parents’ bed and then moved to their own, unresponsive in the morning
2	Methadone	0.19 mg/L	None	The child is thought to have accessed unsecured methadone in the home
3	Carfentanil	8.4 ng/mL	Fentanyl	Parents with a history of substance use. Found in own bed by mom. Carfentanil powder found in home
4	Fentanyl	7.6 ng/mL	None	The parent and child were unhoused. Sleeping on a mattress on the floor with drug-associated material nearby or adjacent
5	Fentanyl	>40 ng/mL	Morphine Cocaine Benzoylecgonine	Parents prescribed methadone. Unclear history, found deceased under living room table. Other substances found on the scene
6	Fentanyl	22 ng/mL	None	Parents prescribed methadone. Bed-sharing with both parents in their bed, found in the morning unresponsive. Other substances found on the scene
7	Fentanyl	18 ng/mL	Etizolam Amphetamine	The parent and two others in the home administered heroin the night before. Found in bed the next morning unresponsive
8	Fentanyl	23 ng/mL	Etizolam	The parent prescribed methadone and administered fentanyl recently. Had a nap in parent’s bed, found to be unresponsive after
9	Fentanyl	10 ng/mL	None	Had a nap on parents’ bed, found unresponsive 1 h after, no drug associated material seen on site
10	Fentanyl	11 ng/mL	None	Napped with a person providing childcare, found unresponsive after. Quantity of fentanyl found outside bedroom

Most of the cases were attributable to substances found in the child’s play or sleeping area, though one case was related to improper storage of prescription medication. A common narrative reported in five cases was a child sleeping in their parent’s bed and being found unresponsive.

## DISCUSSION

We found 10 deaths in the children over the study period. Although it was not the aim of the study, we did examine the 4 years prior to our inclusion criteria and found that the number of opioid-related deaths in the same population was four. This study highlights the staggering effect that opiate use is having on pediatric mortality in the context of drug overdose, correlated with American studies showing a sharp increase in opiate-related deaths (5,10). A US report by Gaw et al. examining fatal poisoning in children 5 years of age or less, found opioids accounted for 24.1% of deaths in 2005 and 52.2% in 2018 while in subsequent work Gaither et al., found that when examining the same age group there was nearly a sixfold increase in opioid-related pediatrics deaths in 2021 compared with 1999 (5,10). This rise is not surprising since previous data showed a positive correlation between adult opioid prescriptions and pediatric opioid mortality; however, the magnitude of this rise is sobering (11). Thus, this sharp incline is being seen throughout the literature without enough emphasis on the circumstances and possible areas of intervention.

The impact of deaths within this age group is significant, particularly due to their frequently preventable nature notably given the exploratory nature of children at the ages seen in our study. Thus, the environments of death in these are of the utmost importance. Our results are unique since a CWS was involved with all cases prior to the child’s death. In comparison, Gaw et al. (10) found only 16.7% of fatal pediatric poisonings had prior CWS involvement. This suggests there is an opportunity for intervention around opioid use and storage to reduce the risk of overdose for children in the home.

A more recent study from North Carolina in 2012 described 19 pediatric deaths related to opioids, primarily among children aged 2 years or younger. They found that 7 of 19 were due to illicit fentanyl and 3 due to fentanyl patches. Similar to our results, 5 of the 7 illicit fentanyl deaths had fentanyl found in the child’s environment. Additionally, many of these cases involved the infant sleeping in the parent’s bed (7). These are comparable circumstances to the ones seen in our patient population suggesting specific patterns including fentanyl as the primary causative agent, drug-associated material in the house, deaths occurring in the home, and children sleeping in parents’ beds or napping.

Utilizing these findings, one could consider possible interventions and areas of public health reform such as increased awareness and access to naloxone through substance use and addiction programs (12). Of note, pediatric pain management standards do not appear to be an issue as no child died due to an opioid prescription of their own. Also, more accurate reports of opioid-related deaths by ranges other than 0 to 19 years would be helpful to define target populations for intervention (12).

Other than ongoing naloxone education and more accurate statistics there are other areas of intervention. These could include CWS home visits, and should drug-associated material be found in the household, action by CWS workers should be taken forthwith and at the minimum require education on access limitation for drug storage and use and follow-up visits to ensure that these steps are put into place. It would also be beneficial for further education related to sleeping practices and safe sleep. US data suggests that between states only the Good Samaritan Law was associated with a reduction in pediatric mortality rate, which is universal across Canada (2).

Limitations to our study are that it is unique to Ontario and that there was some missing data. This highlights the need for further research especially at a national level to better understand trends in Canada. The Canadian province of British Columbia also presents a unique research opportunity on the impact of pediatric mortality of the recent decriminalization of small amounts of opioids and the subsequent reversal of that policy.

In 4 years, 10 children died from opioid-related death in Ontario, most of these were secondary to fentanyl and were preventable notably given that all cases occurred while children were under the mandate of CWS. Proportional to population this is a significant number and should emphasize the need for robust and accountable approaches to identify at-risk children and homes and to put policies in place to protect these children.
